# Novel Histopathological Patterns in Cortical Tubers of Epilepsy Surgery Patients with Tuberous Sclerosis Complex

**DOI:** 10.1371/journal.pone.0157396

**Published:** 2016-06-13

**Authors:** Angelika Mühlebner, Jackelien van Scheppingen, Hanna M. Hulshof, Theresa Scholl, Anand M. Iyer, Jasper J. Anink, Ans M. W. van den Ouweland, Mark D. Nellist, Floor E. Jansen, Wim G. M. Spliet, Pavel Krsek, Barbora Benova, Josef Zamecnik, Peter B. Crino, Daniela Prayer, Thomas Czech, Adelheid Wöhrer, Jasmin Rahimi, Romana Höftberger, Johannes A. Hainfellner, Martha Feucht, Eleonora Aronica

**Affiliations:** 1 Department of Pediatrics, Medical University Vienna, Vienna, Austria; 2 Department of (Neuro) Pathology, Academic Medical Center, Amsterdam, The Netherlands; 3 Department of Pediatric Neurology, Brain Center Rudolf Magnus, University Medical Center Utrecht, Utrecht, The Netherlands; 4 Department of Clinical Genetics, Erasmus MC, Rotterdam, The Netherlands; 5 Department of Pathology, University Medical Center Utrecht, Utrecht, The Netherlands; 6 Department of Pediatric Neurology, Charles University, Second Medical School, Motol University Hospital, Prague, Czech Republic; 7 Department of Pathology and Molecular Medicine, Charles University, Second Medical School, Motol University Hospital, Prague, Czech Republic; 8 Shriners Hospital Pediatric Research Center and Department of Neurology, Temple University, Philadelphia, United States of America; 9 Department of Radiology, Medical University Vienna, Vienna, Austria; 10 Department of Neurosurgery, Medical University Vienna, Vienna, Austria; 11 Institute of Neurology, Medical University Vienna, Vienna, Austria; 12 Swammerdam Institute for Life Sciences, Center for Neuroscience, University of Amsterdam, Amsterdam, The Netherlands; 13 Stichting voor Epilepsie in Nederland (SEIN), Hemstede, The Netherlands; Nathan Kline Institute and New York University School of Medicine, UNITED STATES

## Abstract

Tuberous Sclerosis Complex (TSC) is a genetic hamartoma syndrome frequently associated with severe intractable epilepsy. In some TSC patients epilepsy surgery is a promising treatment option provided that the epileptogenic zone can be precisely delineated. TSC brain lesions (cortical tubers) contain dysmorphic neurons, brightly eosinophilic giant cells and white matter alterations in various proportions. However, a histological classification system has not been established for tubers. Therefore, the aim of this study was to define distinct histological patterns within tubers based on semi-automated histological quantification and to find clinically significant correlations. In total, we studied 28 cortical tubers and seven samples of perituberal cortex from 28 TSC patients who had undergone epilepsy surgery. We assessed mammalian target of rapamycin complex 1 (mTORC1) activation, the numbers of giant cells, dysmorphic neurons, neurons, and oligodendrocytes, and calcification, gliosis, angiogenesis, inflammation, and myelin content. Three distinct histological profiles emerged based on the proportion of calcifications, dysmorphic neurons and giant cells designated types A, B, and C. In the latter two types we were able to subsequently associate them with specific features on presurgical MRI. Therefore, these histopathological patterns provide consistent criteria for improved definition of the clinico-pathological features of cortical tubers identified by MRI and provide a basis for further exploration of the functional and molecular features of cortical tubers in TSC.

## Introduction

Tuberous Sclerosis Complex (TSC) is a genetic disease affecting about 1:6,000 live births [1]. TSC is characterized by an age-dependent manifestation of primarily non-malignant tumors in many different organ systems [2]. In TSC, almost 90% of the affected individuals will suffer from recurrent seizures [3], and thus most patients are diagnosed after the onset of seizures in infancy or early childhood [2]. Only about a third of patients can be treated successfully with anti-epileptic drugs. In the remaining individuals, resective surgery of the corresponding epileptogenic zone (EZ) may be considered [4]. Defining the EZ is a major challenge in TSC patients since the EZ might not be restricted to one single tuber. Therefore, selected patients often undergo intracranial electroencephalography (EEG) evaluation to localize accurately the EZ and eloquent cortex prior to the determination of the resection area. This procedure is an additional risk factor and burden to patients [5]. After epilepsy surgery, 57% of patients achieve seizure freedom and another 18% show a significant reduction in seizure frequency at a minimum of one year follow-up [6]. In addition to seizure freedom, psychomotor development may also improve [6]. However, approximately 25% of patients have unfavorable outcomes after surgery, with ongoing seizures and, in about 3% of cases, major surgical morbidity [7]. Therefore, it is urgent to identify those TSC patients who will significantly benefit from epilepsy surgery, and hence, there is a great need for a clear-cut definition of the EZ, based on imaging-, neurophysiological-, and source localization techniques.

TSC is caused by a mutation of either of two genes, *TSC1* and *TSC2*, which encode the proteins TSC1 (hamartin) and TSC2 (tuberin), respectively [3]. These proteins are involved in numerous regulatory processes via the regulation of the mTOR signaling pathway including cell growth, proliferation, migration and differentiation. In the first reported case series about 90% had cerebral manifestations, including cortical tubers [8]. Histologically, cortical tubers present with a distorted cortical architecture, and contain dysmorphic neurons with aberrant Nissl substance and bright eosinophilic giant cells. It has been proposed that these aberrant cells play an essential role in epileptogenesis [9]. However, recent data from intracranial recordings indicate that the perilesional cortex also plays an important role [10].

We examined cortical tubers and perituberal cortex samples using multiple biomarkers from TSC patients who previously underwent epilepsy surgery as a strategy to classify the histological severity of tubers and potentially aid with future evaluation of the EZ in TSC patients. Using quantitative histology and a dedicated imaging program, we assessed a number of cellular features, including mTORC1 activation, amount of neurons, dysmorphic neurons, calcification, gliosis, giant cells, vessels, inflammatory markers, myelin content and amount of oligodendroglial cells. The results were cross-referenced to clinical data, and analyzed statistically.

## Material & Methods

### Subjects

We critically evaluated 28 tubers from whom we received anatomically well preserved, *en bloc* resected neocortical tissue and sufficient clinical data (Department of Pediatric Neurology, Brain Center Rudolf Magnus, University Medical Center Utrecht; Department of Pediatric Neurology, Charles University, 2^nd^ Medical School, Motol University Hospital, Prague and Department of Pediatrics, Medical University Vienna; median age at resection = 6.00 years; range = 0.83–47 years; localization: 17 frontal, 8 temporal and 3 parietal; gender: 17 males, 11 females). Extensive presurgical evaluation including 24hours to 5 days video-EEG monitoring, high-resolution MRI and neuropsychological testing was performed in each patient in order to characterize the EZ, and to select candidates for tailored surgical resection. We included also 7 perituberal samples which were defined by absence of dysmorphic neurons and giant cells on histology (whole tissue blocs to ensure equality in available grey and white matter).

The age- and localization-matched control group consisted of 23 autopsy cases (median age = 2.00 years; range = 0.1–17 years; localization: 7 frontal, 9 temporal, 7 occipital; gender: 10 males, 13 females; post mortem delay = 24h). None of these patients had a history of seizures or other neurological diseases. All control samples have been collected at the Department of (Neuro)Pathology, AMC, Amsterdam, The Netherlands.

Tissue was obtained and used in accordance with the Declaration of Helsinki and the AMC Research Code provided by the Medical Ethics Committee and approved by the science committee of the UMC Utrecht Biobank. This study was also approved by the Ethical Committee of the Medical University of Vienna and the Ethical Committee of the Motol University Hospital in Prague. Written informed consent was obtained from all patients included into our study.

### Tissue preparation and staining protocols

The tissue was carefully oriented, cut perpendicular to the pial surface, fixed overnight in 4% formaldehyde and routinely processed into liquid paraffin. Sections were cut at 4–6 μm with a microtome (Microm, Heidelberg, Germany), and mounted on positively charged slides (Superfrost + Menzel, Germany). Each specimen was histopathologically examined using haematoxylin & eosin (H & E). An immunohistochemical examination of all surgical specimens was performed using the following panel of antibodies: Olig2 (oligodendrocyte lineage transcription factor 2, 1:100 dilution, IBL, Minneapolis, USA), NeuN (neuronal nuclei, 1:100, clone A60, Chemicon, Billerica, MA, USA), non-phosphorylated neurofilament H (1:1000, clone SMI32, Sternberger, Lutherville), GFAP (glial fibrillary acidic protein, 1:4000, Dako, Glostrup, Denmark), CD3 (cluster of differentiation 3, 1:200, clone F7.2.38, DAKO), HLA-DP, DR, DQ (human leukocyte antigen class II, 1:100, clone Cr3/43, DAKO), pS6 (phosphorylated S6-ribosomal protein, 1:1200, Ser235/236, Cell Signalling Technology, Danvers, MA, USA), MBP (myelin binding protein, 1:400, DAKO), CD34 (cluster of differentiation 34, 1:600, Qbend, Immunotech) and vimentin (1:500, clone V9, Dako). Autopsy cases were pre-treated with 1% Triton X-100 (for 1 h) prior to incubation with anti-Olig2 antibody. The slides were air dried overnight at 37°C. All immunohistochemical stainings were performed with a Ventana semiautomated staining machine (Benchmark ULTRA; Ventana, Illkirch, France) and the Ventana DAB staining system according to the manufacturer’s protocol.

### Semi-quantitative measurements

Slides were scanned with an Olympus dotSlide system (vs 2.5, Olympus, Tokyo, Japan). Digital slide scans were obtained at a 100x magnification with a resolution of 0.64μm/pixel. Scans were exported as a homogeneous set of.TIFF files, each with an equal image size of 50MB. Files without visible tissue were discarded. Qualitative and semi-quantitative image analysis was performed with the Image-Pro Premiere software package v. 9.1 (Media Cybernatics, USA). The mean and standard deviation was calculated for every parameter and used for statistical analysis.

### Calcifications

The presence or absence of calcifications was scored based on the H&E staining This was converted into a nominal variable of 0 and 1.

### Giant cells

The numbers of giant cells per mm^**2**^ were calculated from 10 representative fields (each representing 1.081mm^**2**^) of an anti-vimentin stained section.

### Microglial activation, gliosis, mTORC1 activation and myelin content

Quantification of all available tissue was taken into account. In a first step the total tissue surface area was calculated for each case utilizing the smart segmentation tool provided by the ImagePro software package. Second, the DAB positive area (anti-Cr3/43, anti-GFAP, anti-pS6-Ser235/236 and anti-MBP) was separated from background using an adjusted protocol for segmentation (S1 Fig). All stacks of images were submitted to a batch processing algorithm that was kept consistent throughout the whole analysis. In a final step, the overall percentage of positivity was assessed for each case and used for statistical analysis. In the myelin stainings we further addressed the optical density (OD) of the MBP positive area. The integrated OD of MBP was also used. For simplicity and due to the linear correlation (p < 0.001), the product of intensity x frequency (percentage x OD) was calculated. This product is further referred to as overall myelin content (OMC).

### Cellular densities

For the calculation of cellular densities the available images were split into RGB channels. To establish the positive count a brightness threshold in the blue channel was determined for each staining (NeuN: 120; CD3, SMI32 and Olig2: 100; CD34: 150) and size ranges were defined to allow more accurate counts (pixel areas: NeuN: 120–2500; CD3: 20–155; Olig2: 20–155 in surgical tissue and 5–120 in autopsy material; CD34: 100-unlimited; SMI32: 750–5000). These parameters were kept the same throughout the analysis and were also implemented in the batch processing algorithm. All cellular densities are represented as total number/mm^**2**^.

### Clinical data

All available MRIs were retrospectively reviewed and the resected tubers were scored according to the classification system proposed by Gallagher et al. [11]. The presence of “focal cortical dysplasia (FCD)-like” features (thickened cortex and blurred grey-white matter border in areas surrounding one or several tubers and presence of transmantle sign) were assessed as well as the presence of calcifications. In addition, the following clinical data was obtained: *TSC1/TSC2* mutation status, gender, age at seizure onset, localization of the resected area, age at epilepsy onset, mean seizure frequency (daily-weekly-monthly), antiepileptic drug (AED) management at surgery, type of epilepsy surgery, duration of active epilepsy, last available postsurgical seizure outcome (according to Engel’s score), average intelligence quotient (IQ) with global cognitive performance and presence/absence of autism [12].

### Interobserver agreement

For case evaluation, six different neuropathologists gained access to an online virtual slide system (Digital Slidebox 4.5, Slidepath; Leica Microsystems, Dublin, Ireland). These reviewers were asked to classify a subset of ten randomly selected cases provided with three basic stainings (H&E, SMI32 and vimentin) according to the novel scheme of patterns discussed in this manuscript. After 21 days the platform was closed. None of the reviewers had access to the results of the others.

### Statistical analysis

Statistical analysis was performed on SPSS 21 (IBM, PASW Statistics, USA). The distributions of pS6, SMI32 and vimentin were left skewed. Therefore, the data were log-transformed prior to statistical analysis. Hierarchical clustering (Ward’s method with squared Euclidian distances) and one-way ANOVA were used for specify the tuber patterns (S2 Fig). Due to the lack of normality and non-equality of variances non-parametric testing (independent-sample Kruskal-Wallis test) as well as Kendall-tau correlation were used to analyse the data. Partial correlation was applied if data needed to be corrected for another variable. The Chi-squared test was applied for analysing categorical data. The κ coefficient was calculated to address inter-rater variability. In our study κ was interpreted as follows: <0.2, poor agreement; 0.2–<0.4, fair agreement; 0.4–<0.6, moderate agreement; 0.6–<0.8, good agreement; 0.8–1.0, very good agreement. Bootstrapping was conducted on 1000 samples with bias-corrected and accelerated confidence intervals. P-values were considered significant if < 0.05.

## Results

### Patterns of cortical tubers

**Fig 1:**
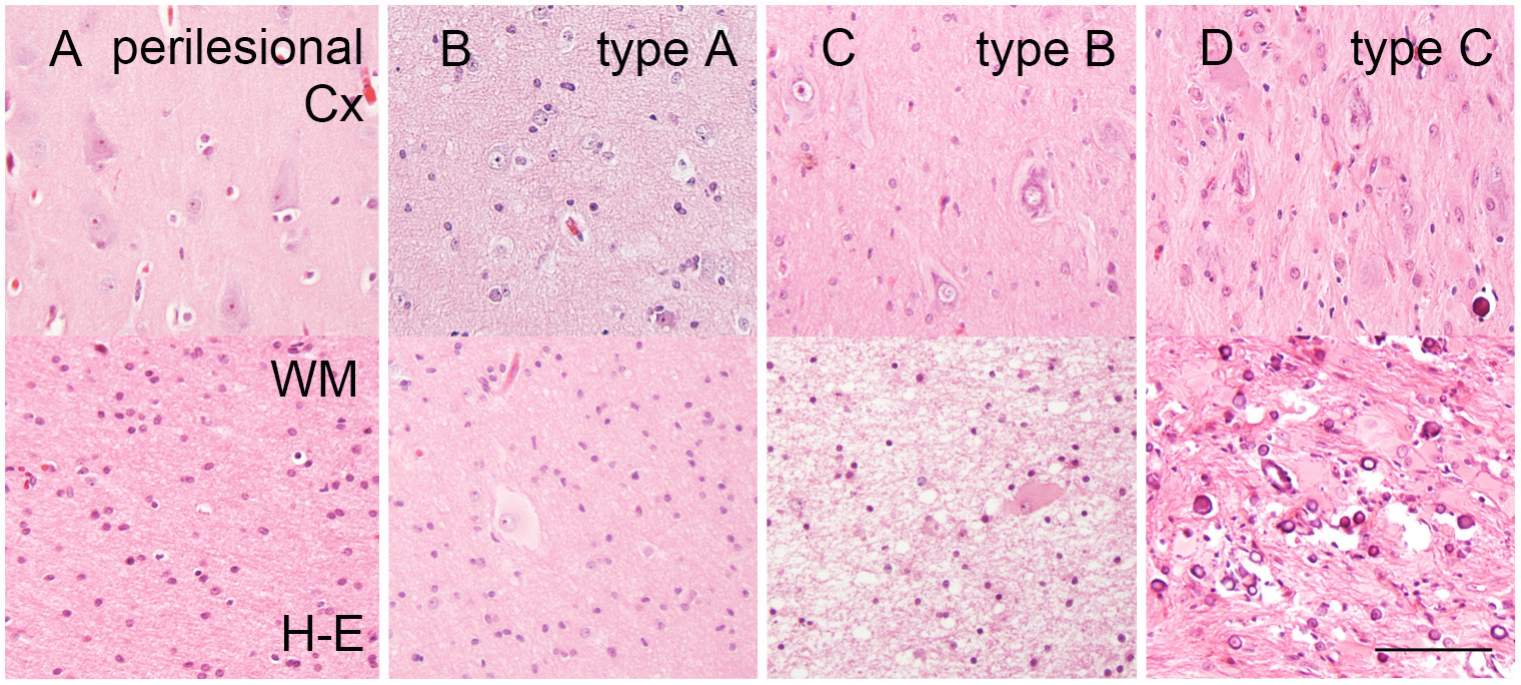
Histology of tuber variants. **A.** Perilesional cortex (Cx) and white matter (WM) of an 8-year old male patient. **B.** Type A tuber (Cx and WM) in a 5-year old female patient with a tuber in the frontal lobe. **C.** Type B tuber (Cx and WM) located in the parietal lobe of an 8-year old boy (same as in Fig 1A). **D.** Type C tuber of a 2-year old male patient located in the frontal lobe. *Scale bar in D = 100μm and applies also to A*, *B and C*.

In order to find differences in histological appearance the three most accessible features of a cortical tuber (mTORC1 activation, dysmorphic neurons and giant cells) were admitted to a hierarchical cluster analysis. As a result, the tubers were divided into three different clusters (S2 Fig). To determine the main histological discriminants between the clusters a one-way ANOVA was performed. There were significant differences in the numbers of dysmorphic neurons (p = 0.000) and giant cells (p = 0.001) whereas pS6-Ser235/236 positivity failed to reach significance. Tubers were then independently analyzed by two neuropathologists experienced in evaluating epilepsy surgery specimens (Angelika Mühlebner and Eleonora Aronica) and the microscopic assessment of the clusters revealed three different patterns. These were translated into the following qualitative criteria:

1. low density of giant cells ≤ 10/mm^**2**^ or dysmorphic neurons ≤ 3/mm^**2**^ (Fig 1B)
2. high density of giant cells > 10/mm^**2**^ or dysmorphic neurons > 3/mm^**2**^ (Fig 1C)
3. giant cells, dysmorphic neurons and calcifications (Fig 1D)

We submitted a randomly selected subset of cases to an online platform in order to validate our findings among other trained neuropathologists (six participants). The inter-observer agreement was very good (κ = 0.973).

The following quantitative analysis was based on 28 available tuber specimens. Subsequently, our cohort consisted of 7 type A tubers (median age = 10.00 years, range 3–47 years; 6 males, 1 females; 5 frontal, 2 temporal), 13 type B tubers (median age = 7 years, range 0.83–22 years; 6 males, 7 females; 4 frontal, 6 temporal, 2 parietal, 1 hemispheric) and 8 type C tubers (median age = 3 years, range 1–6 years; 5 males, 3 females; 7 frontal, 1 parietal).

### mTORC1 activation, neuronal quantity and gliosis

**Fig 2:**
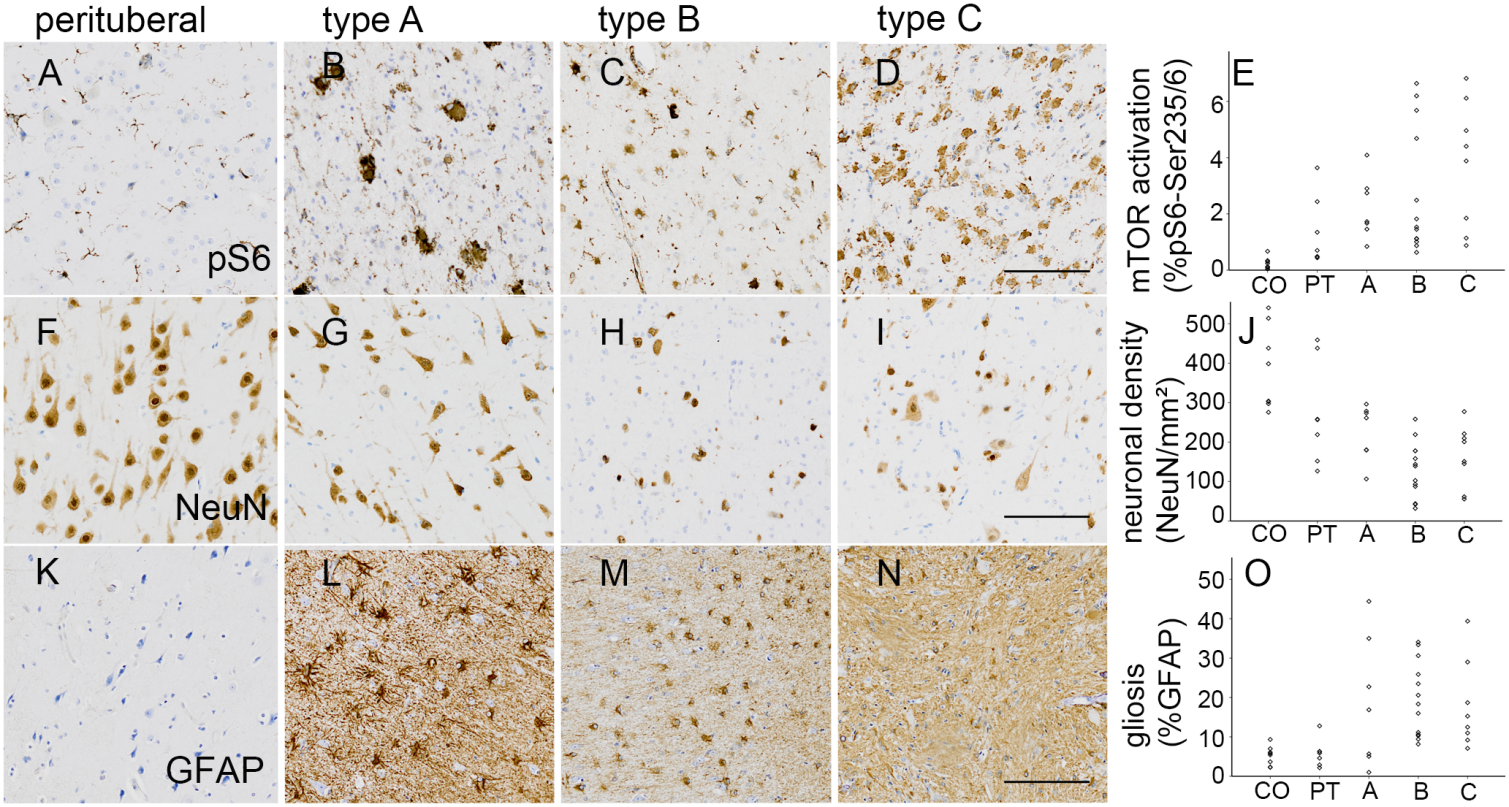
mTORC1 activation, neuronal quantity and gliosis. **A.—D.** mTOR activation was present in all cortical tuber types as well as in perituberal cortex (pS6-Ser235/236 staining). **E.** Gradient mTOR activation among the tuber types. **F.—I.** Loss in neuronal cell density could be observed in type B tubers (NeuN staining). **J.** Neurons were significantly depleted (*p = 0*.*015*). **K.—N.** Gliosis was present in all tuber types (GFAP staining). **O.** Increase of gliosis reached significance (*p = 0*.*019*). *Scale bar in D*, *I*, *and N = 100μm and applies also to F*, *G*, *H*, *K*, *L*, *and M*. *CO = control; PT = perituberal cortex*.

In our cohort we compared with histologically normal cortex to clarify the role of perituberal cortex. Independent-sample Kruskal-Wallis testing revealed no differences in mTORC1 activation among the tuber types and perilesional samples (Fig 2A–2E). However, mTORC1 activation was significantly increased in all samples compared to controls (H[4] = 22.942, p = 0.000). Interestingly, in the distribution of NeuN positive cells/mm^**2**^ varied significantly among the subgroups (H[3] = 10.416, p = 0.015; median and range of all quantification data see Table 1). However, subsequent pairwise comparison did not reveal significant changes. We could however observe a tendency in type B tubers to show decreased neuronal cell counts (Fig 2F–2J). Quantification of GFAP positivity revealed a variation in distribution among the groups (H[3] = 9.943, p = 0.019; Table 1). Pairwise comparison showed a significant increase of GFAP density in type B tubers when compared to perituberal samples (p = 0.023). All calculations were based upon 28 tuber samples and 7 perituberal cortex specimens. Subgroup analysis revealed no correlation of mTORC1 activation, neuronal density or gliosis with the localization of the tuber. However, the amount of GFAP positivity was negatively correlated with the age at surgery (Kendall-tau, R = -0.271, p = 0.025).

**Table 1:** Quantification.

Classification						
Median (range)	type A	type B	type C	Perituberal	control	p-value
**pS6 (%)**	**1.73 (0.84–4.09)**	**1.53(0.63–6.64)**	**4.14(0.88–6.82)**	**0.70(0.44–3.64)**	**0.18 (0.03–0.67)**	**0.000**
**neurons/mm**^**2**^	**261.20(106.56–296.26)**	**139.18(32.03–258.29)**	**175.88(55.70–277.31)**	**256.63(126.69–459.07)**	**351.39(275.69–540.46)**	**0.015**
**GFAP (%)**	**16.87(1.01–44.41)**	**18.34(8.16–34.06)**	**13.87(7.09–34.02)**	**5.89(2.15–12.78)**	**5.61(2.25–9.34)**	**0.019**
**T-cells/mm**^**2**^	**7.70(4.23–19.32)**	**13.81(7.38–35.85)**	**22.64(7.29–34.20)**	**9.08(1.91–13.55)**	**5.54(0.56–29.76)**	**0.002**
**Cr3/43 (%)**	**3.10(0.21–7.63)**	**2.16(0.25–33.24)**	**5.14(0.26–11.66)**	**.95(0.11–2.50)**	**.51(0.07–1.70)**	**0.003**
**vessels/mm**^**2**^	**73.85(36.65–169.35)**	**73.90(40.06–190.45)**	**114.48(77.57–154.12)**	**66.93(28.49–114.06)**	**130.41(36.77–239.79)**	**0.012**
**OMC (%lum)**	**389.95(161.97–3185.74)**	**196.23(0.24–8110.52)**	**273.460.01–3168.69)**	**2711.58(909.27–6869.83)**	**2801.68(188.05–5815.44)**	**0.020**
oligos/mm^**2**^	159.22(9.51–405.16)	103.03(20.51–390.42)	133.31(66.73–832.46)	184.98(109.86–334.99)	208.17(88.01–522.37)	>0.05

All available quantification data of the image analysis study. Significance levels are presented in the last column.

To broaden our knowledge of these novel patterns we subsequently introduced an age- and region-matched control group to see whether the subgroups differ from autopsy controls.

### Inflammation and small vessel density

**Fig 3:**
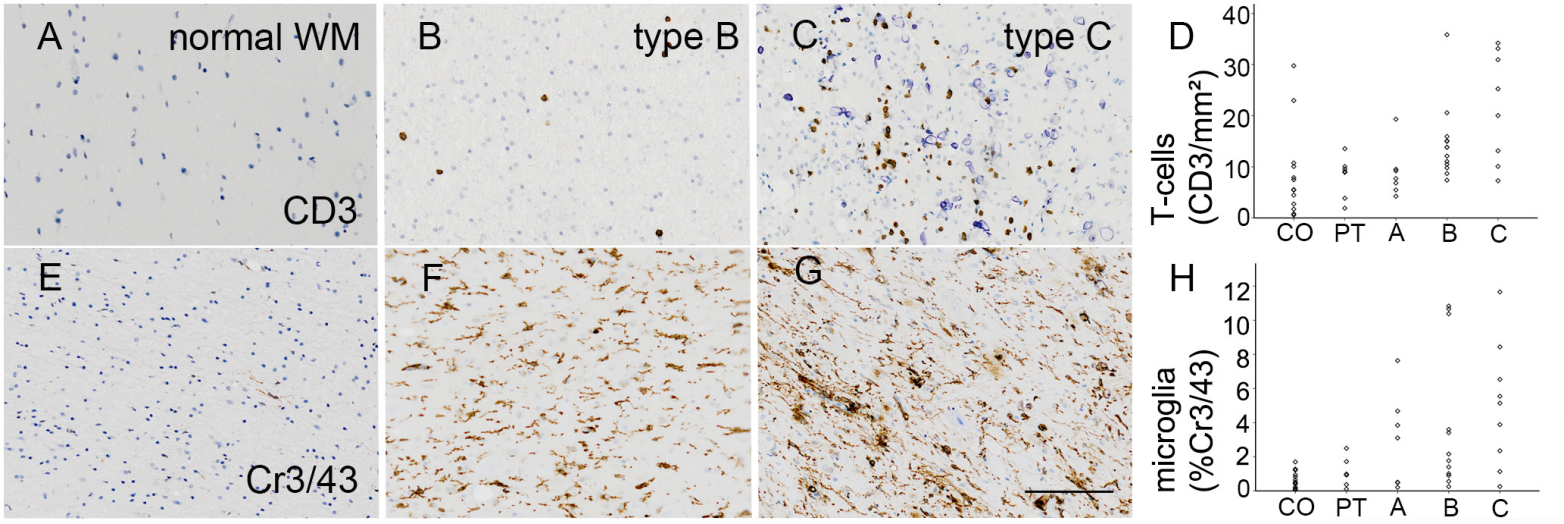
Inflammatory markers and vessel structure. **A.** Representative amount of T-cells lying in the white matter (WM) of control samples (CD3 staining). **B. + C.** T-cell content within type B and C tubers (CD3). **D.** Significant increase of CD3 positive cells in type B and C tubers compared to controls. **E.** Almost no microglial activation can be detected in autopsy cortex (Cr3/43 staining). **F. + G.** Representative WM of type B and C tubers showing an increase in microglial activation (Cr3/43). **H.** Quantification of the Cr3/43 positive content revealed a significant difference between controls and type B as well as C tubers. *Scale bar* in *g* = 100μm and applies also to *a*, *b*, *c*, *e*, *f and g*. *CO = control; PT = perituberal cortex*.

An increase in inflammatory cells in cortical tubers has been previously shown [13]. The amount of T-cells (CD3 positive cells/mm^**2**^) was significantly affected by the tuber type (H[4] = 16.730, p = 0.002; Table 1). We confirmed a gain of inflammatory markers in type B and C tubers compared to postmortem control in a pairwise comparison (B: p = 0.047; C: p = 0.012; Fig 3A, 3B and 3C). A similar pattern was be observed in microglial activation (percentage of Cr3/43 positivity; H[4] = 16.417, p = 0.003; Table 1; Fig 3E–3G). Type B and C tubers showed a significant gain in microglial activation (B: p = 0.016; C: p = 0.005) compared to control (Fig 3H). No differences could be detected for type A tubers. Furthermore, we were able to detect a difference in small vessel density (CD34 staining) among the categories (H[4] = 12.835, p = 0.012; Table 1). However, pairwise comparison failed to reach significance when directly compared to autopsy brain specimens.

### Myelin content and oligodendroglial cell count

**Fig 4:**
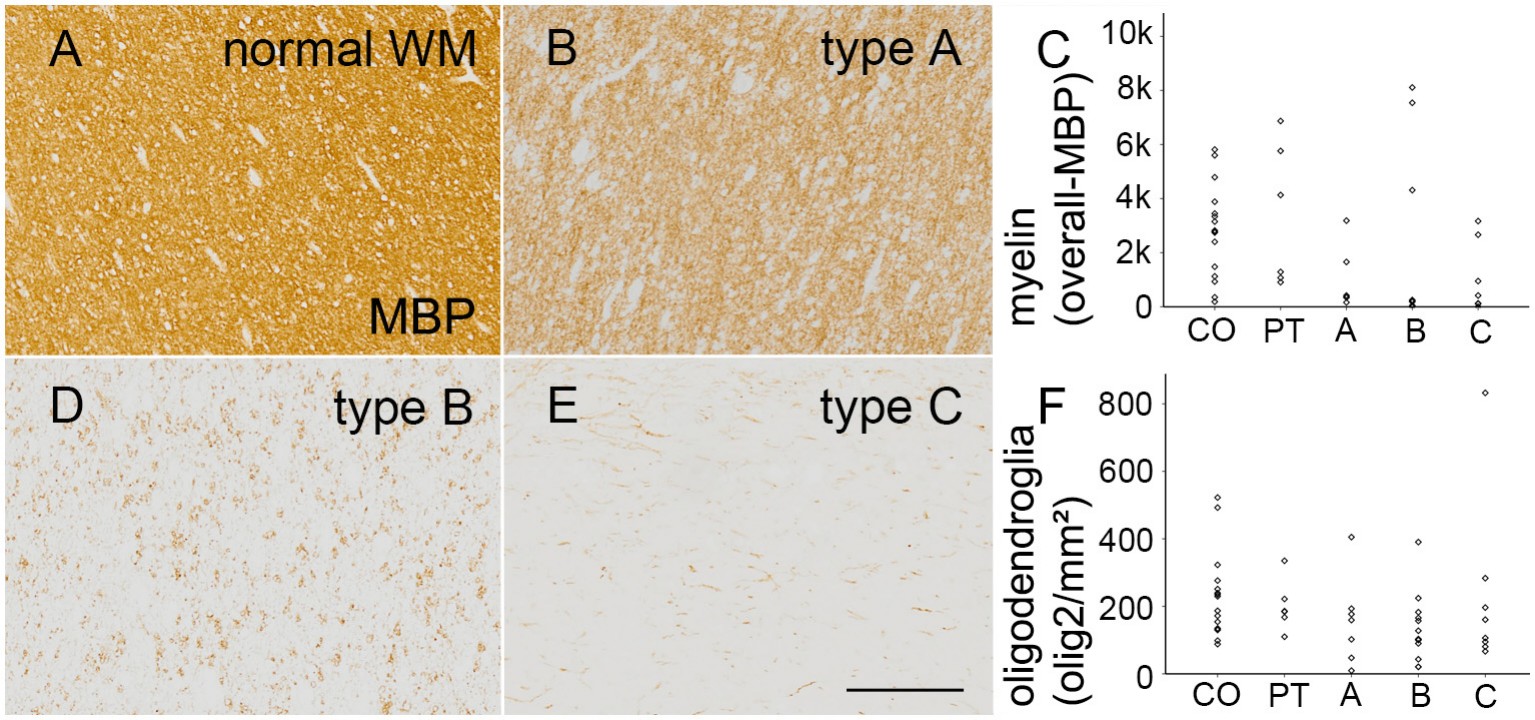
White matter pathology in cortical tubers. **A.** Normal appearing white matter content of the temporal lobe in a 17-years old autopsy case (MBP staining). **B. + D. + E.** Representative sections of WM within the 3 different tuber types (MBP). *Scale bar* in e = 100μm and applies also to *a*, *b*, *and d*. **C.** Significant reduction of myelin content. **F.** Equal distribution of oligodendroglial cells among all tuber types. *CO = control; PT = perituberal cortex*.

Within the tuber patterns we were able to observe myelin loss whereas there was no difference in oligodendroglial cell count (Fig 4A, 4B, 4D and 4E). The overall myelin content (OMC, based on MBP) was altered among the different categories (H[4] = 11.691, p = 0.020; Table 1; Fig 4C).

Neither OMC nor the number of olig2 positive cells were related to age or localization in controls. There was no significant difference between autopsy and perituberal samples.

### Correlations with clinical data

**Fig 5:**
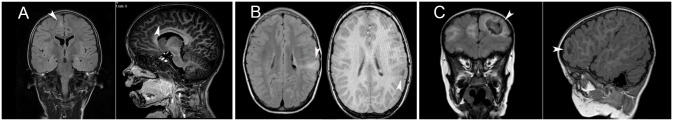
Clinical implications. **A.** Representative MRI of a patient with a histological type A tuber (histology is shown in Fig 1B) characterized by a hyperintense lesion parasaggital in the right frontal lobe (indicated by the arrow head) on T2 weighted and fluid-attenuated inversion recovery (FLAIR) and a hypointense signal in the same area on volumetric T1 images. **B.** MRI of a histological type B tuber (histology is shown in Fig 1C) with FCD-like features in the left postcentral/parietal region, showing as a hyperintense lesion on FLAIR image and a hypointense signal on 3DT1. FCD features are recognized by thickened cortex, blurring of gray and white matter junction and a transmantal sign. **C.** MRI of a histological type C tuber (histology is shown in Fig 1D) with a large calcification, characterized by deep hypointense signal with surrounding heterogenous hyperintense signal on T2 weighted and FLAIR images and a hypointense signal in the white matter on 3DT1. The above described FCD features are seen here as well.

There was no relation between the tuber types according to Gallagher et al. [11] detected on MRI and the histological tuber classification on the sequences available (Fig 5A–5C). However, FCD-like features assessed on presurgical MRI were significantly related with histological type B and C tubers (X^**2**^; p = 0.037). Interestingly, a lower drug load (measured by the number of antiepileptic drugs [AED] taken at the time of the surgery) was associated with type A tubers (X^2^, p = 0.011). Furthermore, carbamazepine, levetiracetam, valproic acid, topiramate, clobazam and vigabatrine were the drugs of choice in this specific group, never reaching a combination of more than two given at the time of surgery. In the other subtypes various range of all available drugs in combination of up to five were found. Hemispherotomies were not observed in the type A group. In addition, we observed a negative correlation between histological tuber type and age at surgery as well as duration of active epilepsy (Kendall-tau and partial; age: p = 0.002; epilepsy duration: p = 0.004). All other clinical characteristics failed to reach significance (Table 2).

**Table 2:** Patients characteristics.

	Classification			
	type A	type B	type C	p-value
Gender				
male	6	6	6	
female	1	7	2	> 0.05
localization				
frontal	5	5	7	
temporal	2	6	0	
parietal	0	2	1	
occipital	0	0	0	> 0.05
seizure frequency prior to surgery				
daily	7	10	6	
weekly	2	3	0	> 0.05
TSC mutation				
tested/ not found	0	1	0	
TSC1	1	5	2	
TSC2	5	3	2	
not tested	6	4	4	> 0.05
types of seizures at surgery				
infantile spasms	2	3	1	
focal seizures with awareness	2	5	6	
focal seizures with impaired awareness	3	5	1	> 0.05
MRI classification according to Gallagher et al. 2010 [11]				
A	1	1	0	
B	2	3	3	
C	0	1	1	
combination	4	2	2	
not applicable	0	4	2	> 0.05
**FCD-like features**				
**no**	**4**	**4**	**3**	
**yes**	**3**	**7**	**5**	**0.037**
Side				
left	1	6	3	
right	6	7	5	> 0.05
type of surgery				
lesionectomy	6	7	5	
lobar resection	1	3	1	
hemispherotomy	0	3	2	> 0.05
last known ENGEL score [12]				
1	3	10	4	
2	2	0	1	
3	1	0	2	
4	1	3	1	> 0.05
global cognitive impairment				
none	3	4	0	
mild	1	5	2	
moderate	0	2	3	
severe	3	2	3	> 0.05
IQ	72.50 (45–102)	59(48–107)	52(34–68)	> 0.05
Autism				
no	5	9	4	
yes	2	3	4	> 0.05
**median age at surgery**				
**(range)**	**10.00 (3.00–47.00)**	**7.00 (0.83–22.00)**	**3.00 (1.00–17.00)**	**0.002**
**median duration of active epilepsy**				
**(range)**	**8.00 (3.00–35.00)**	**4.00 (0.80–13.00)**	**2.21 (0.43–13.00)**	**0.004**

All available clinical data were collected and tested for correlation with the histologically classified tuber types. Significance levels are presented in the last column.

## Discussion

Over the past decade a number of studies have been published that focused on the histological features of cortical tubers in TSC patients [14, 15, 16]. However, due to the increased number of patients who underwent epilepsy surgery more and more tissue becomes available to investigate the variability of cellular features within this highly selected patient group. Here, we present the first comprehensive histological analysis with respect to TSC cortical tubers.

Recently, two new classification schemes were presented by task forces of the International League Against Epilepsy (ILAE). In 2011, FCD, the most common cause of intractable epilepsy in children,–- was addressed [17], and in 2013 a novel classification scheme for hippocampal sclerosis was published [18]. These schemes, however, were established to distinguish distinct entities considering clearly different clinical etiologies. Nevertheless, the new FCD classification scheme has already proven to be more reliable with regard to inter-rater variability than previous schemes [19, 20]. Most importantly, the first reports on possible clinical value for prediction of surgical outcome have been published [21, 22]. In these studies, seizure-freedom was dependent on accurate definition of the epileptogenic zone and the subsequent extent of surgical resection [21, 22].

Until now cortical tubers have been neglected in this respect. In an attempt to meet the current need of a better histological assessment we have identified three distinct patterns of cortical tubers. Consequently, we were able to show that our tuber patterns are recognizable by different neuropathologists and therefore reasonably applicable in different neuropathology laboratories. In our endeavor to identify variants of TSC lesions on a histological level and to stay within the frame of diagnostic usefulness and high accessibility we chose a panel of antibodies that has been established and widely-used previously and subsequently used statistic modeling to asses quantitative differences [23]. The normal expression pattern of the selected markers has been also evaluated in post-mortem control tissue. However, one limitation of this study is the availability of brain tissue within the age range of the TSC subjects. An ideal experimental design (including also ages ranging between 1 and 18 year of age) is difficult to achieve and representative material from patients without any significant brain pathology is not available at all developmental ages.

There are only a few studies which report highly advanced methods of quantitative histology on epilepsy surgery specimens [24, 25, 26, 14]. Most of these addressed very specific pathological features to show differences in expression patterns and therefore a lot of the data were generated via a region of interest (ROI) based approach. So far, only one group has applied whole slide scanning and compared fully automated and user-based approaches [27]. In order to access a much broader spectrum of characteristics and obtain a more accurate diagnosis, we choose not to follow a ROI-based approach.

The limitation of choosing to assess all available tissue is the risk to miss subtle changes restricted to only part of the visible pathology. In light of this, our aim was not to specifically assess cellular features but primarily to find distinctive characteristics within the histology. Numerous studies exist to explore the pathogenetic mechanism behind the aberrant cells visible in TSC samples. However, these are often related to FCD Type IIB another pathology with similar histological features [28, 29, 30, 31].

Despite this limitation, it was possible to address several aspects that relate to pathophysiological mechanisms in tubers. In TSC patients mTORC1 activation as assessed by staining for various forms of phospho-S6 is expected due to the genetic nature of TSC. It provides a molecular explanation for the observed giant cells, although the molecular consequences of second-hit events are less well understood. In addition, pS6 has recently been detected immunohistochemically in the perituberal cortex [14] and mTORC1 activation is believed to begin in the fetal period [32, 33]. However, we were not able to detect differences between tuber subgroups when compared to perituberal samples suggesting that also in our study the true margins of the lesion extent beyond the radiographically visible perituber [34].

Furthermore, our data suggests a gradient in the level of mTORC1 activation throughout all tuber types. Our identification of patterns would improve our understanding of the molecular and functional events during tuber pathogenesis and eventually help to find a better definition of perituberal tissue.

In previous studies we demonstrated that inflammation plays an important role in the epileptogenesis of cortical tubers [13, 35]. In the present study we detected increases in inflammatory markers in type B and C tubers. Type A were not significantly different to controls. This observation may argue against the hypothesis that inflammation alone may underlie the epileptogenicity of the tuber. Accordingly, several studies suggest increase neuronal excitability through different mechanisms, including also a deregulation of astrocyte-mediated glutamate uptake and release, as well as through changes in the function of both glutamate and GABA receptors [36]

Furthermore, hypomyelination is a common feature of FCD type II, although it is not fundamental to make the distinction between FCD type IIA and IIB [24, 25]. Notwithstanding, the underlying etiology is less well understood. With our set of data a lack of myelin could also be confirmed. After all, the oligodendroglial cell count did not delineate significant changes compared with controls. Findings in TSC mouse models indicate that there is a marked difference in brain myelination when TSC1 is lost in neurons, suggesting that the reduction in myelination is secondary to mTORC1 activation in neurons, rather than reflecting a primary oligodendroglial abnormality. Also, previous studies sreported that the number of oligodendroglial cells remain intact and that there is axonal loss [25, 14]. Another study suggested that, at least in FCD type IIB there is also a maturational problem [24]. However, the different methodological approaches may have led to these controversial results.

All together certain features of our assessment (neuronal loss, gliosis, calcification, myelin loss and inflammation) are considered regressive changes in pathology in TSC cortical tubers [14, 34], albeit our associations with the available clinical data suggests differently. In our cohort of type A tuber only a limited set of AEDs were prescribed and none of these patients underwent hemispherotomy as surgical strategy. We have to acknowledge that these parameters are certainly not a reflection of electrophysiological properties but they might act as surrogate markers for a milder phenotype. However, in our cohort, the number of patients with type A tuber was too small to evaluate the possible prognostic value on postsurgical seizure outcome or other clinical parameters, which deserves further investigation in prospective studies, using the proposed histological tuber classification.

Another striking feature was the negative association between age at surgery and histology, with type C tuber occurring more often in very young children. This finding could be related to the time point of tuber formation leading to such severe pathological changes already in the early stages of cerebral development [37]. Considering this, calcification, inflammation and increased gliosis in type C tubers may not exclusively indicate regressive changes but also a distinct pathogenesis. Furthermore, the relatively short duration of active epilepsy before surgery is consistent with a more severe phenotype with higher seizure burden and possible comorbid developmental problems. However, our cohort might be too small to detect subtle differences in clinical characteristics in relation to surgical outcomes.

We collected clinical data with special attention to MRI characterization. We were unable to detect an association between the previously proposed classification of tuber types on MRI and our histological assessments [11]. Nevertheless, we identified a correlation between so-called “FCD-like features” and histological type B and C tubers. We can only speculate about the significance of such findings to help identify the epileptogenic zone, but they might give insights into the high similarity of TSC cortical tubers and resected FCD type IIB lesions with regard to their epileptogenicity [38]. It is clear that neuroimaging findings are increasingly relevant in relation to identifying the epileptogenic zone [38, 39, 24].

In summary, we have identified three distinct histological patterns in TSC cortical tubers based on quantitative histological features to increase our understanding of the differences among these lesions, and to lay the groundwork for further research on the functional and molecular effects of the different tuber types.

## Supporting Information

S1 Fig Colour segmentation recipe. For extraction of the total amount of DAB staining the following filters were used to calculate the accurate amount.
(TIF)

S2 Fig Cluster analysis. **A.** Hierarchical clustering (Ward’s method) on the parameters: SMI32 (amount of dysmorphic neurons), vimentin (amount of giant cells) and pS6-Ser235/236 (mTOR activation) identified three different tuber patterns. **B.** 3D dot plot of the identified patterns within their clustering matrix.
(TIF)
