# Supplementary figures and images for: Novel Histopathological Patterns in Cortical Tubers of Epilepsy Surgery Patients with Tuberous Sclerosis Complex
Source: PLoS One. 2016 Jun 13;11(6):e0157396. doi: 10.1371/journal.pone.0157396 (PMC4905625; doi:10.1371/journal.pone.0157396)

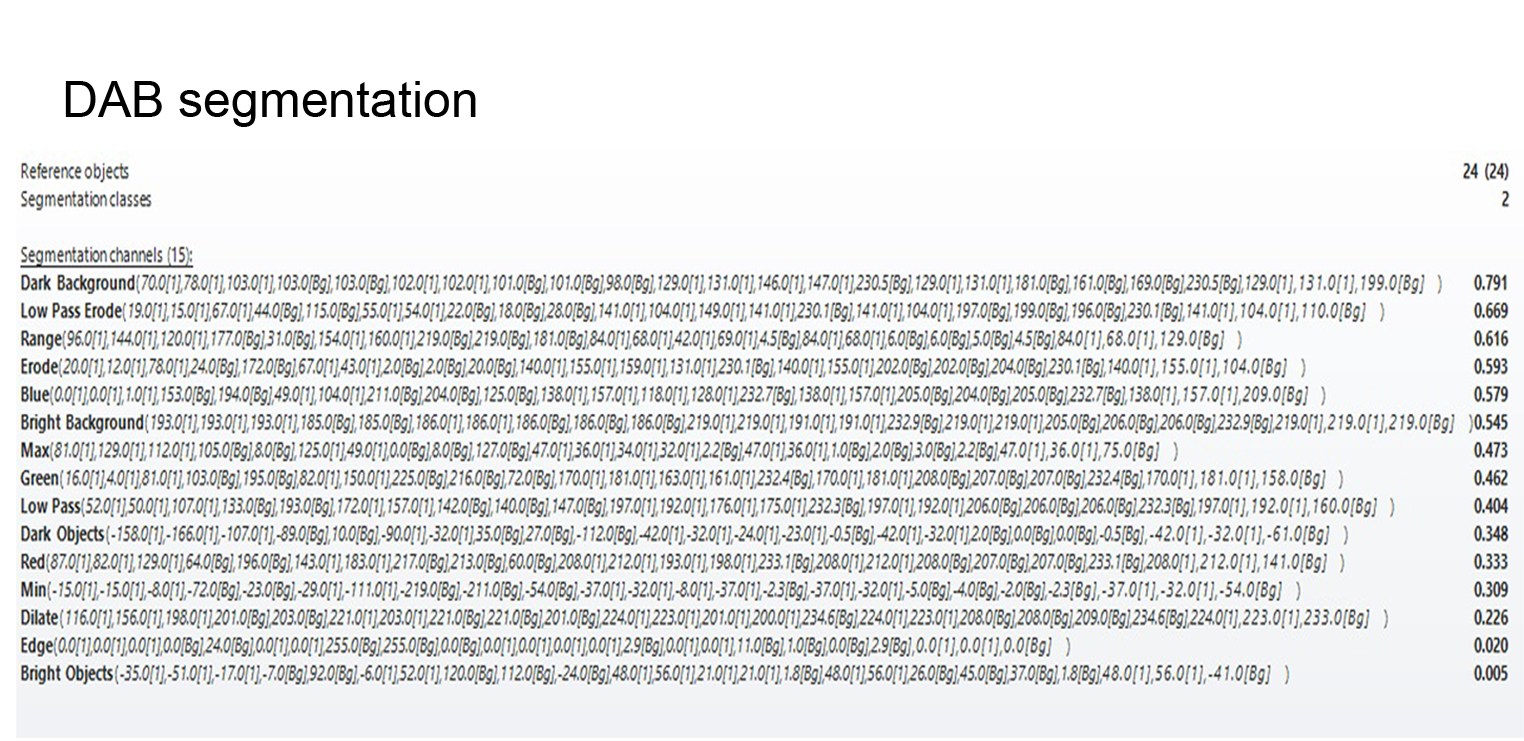

Supplement: S1 Fig — For extraction of the total amount of DAB staining the following filters were used to calculate the accurate amount. (TIF) [file pone.0157396.s001.tif]

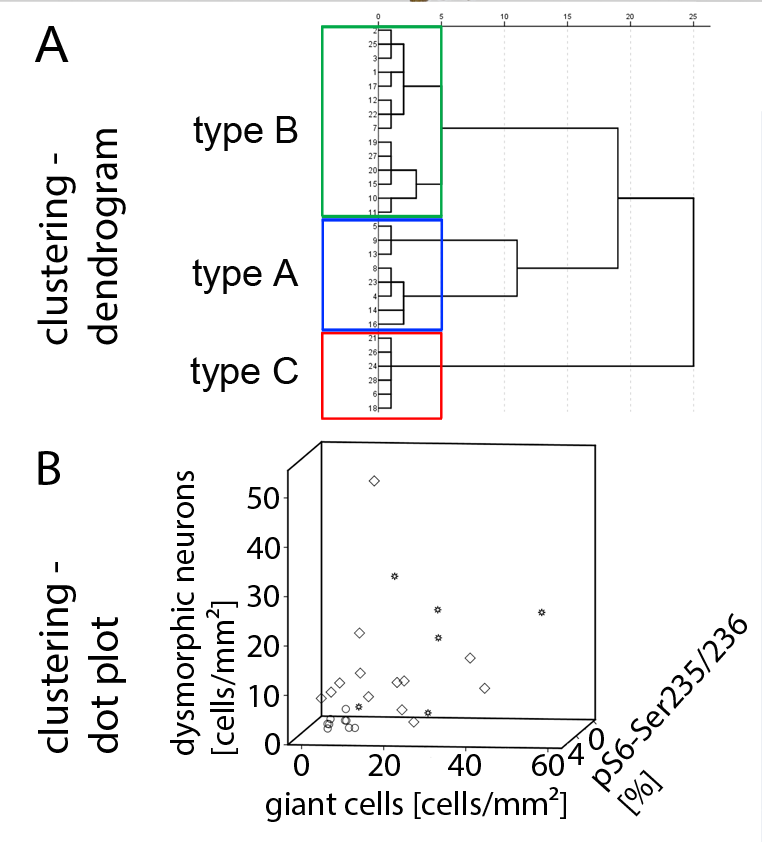

Supplement: S2 Fig — A. Hierarchical clustering (Ward’s method) on the parameters: SMI32 (amount of dysmorphic neurons), vimentin (amount of giant cells) and pS6-Ser235/236 (mTOR activation) identified three different tuber patterns. B. 3D dot plot of the identified patterns within their clustering matrix. (TIF) [file pone.0157396.s002.tif]
